# Lower end of treatment HBsAg and HBcrAg were associated with HBsAg loss after nucleos(t)ide analog cessation

**DOI:** 10.1186/s12876-023-02852-x

**Published:** 2023-06-29

**Authors:** Yandi Xie, Minghui Li, Xiaojuan Ou, Sujun Zheng, Yinjie Gao, Xiaoyuan Xu, Ying Yang, Anlin Ma, Jia Li, Yuemin Nan, Huanwei Zheng, Juan Liu, Lai Wei, Bo Feng

**Affiliations:** 1 Beijing Key Laboratory of Hepatitis C and Immunotherapy for Liver Diseases, Peking University People’s Hospital, Peking University Hepatology Institute, Beijing International Cooperation Base for Science and Technology on NAFLD Diagnosis, Beijing, 100044 China; 2 Department of Hepatology Division, Beijing Ditan Hospital, Capital Medical University, Beijing, 100015 China; 3 Liver Research Center, Beijing Friendship Hospital, Capital Medical University, Beijing, 100050 China; 4 Complicated Liver Diseases and Artificial Liver Treatment and Training Center, Beijing Municipal Key Laboratory of Liver Failure and Artificial Liver Treatment and Research, Beijing Youan Hospital, Capital Medical University, Beijing, 100069 China; 5 Department of Infectious Diseases, The Fifth Medical Center, General Hospital of PLA, Beijing, 100039 China; 6 Department of Infectious Diseases, Peking University First Hospital, Beijing, 100034 China; 7 Department of Infectious Diseases, The Second Hospital of Xingtai, Xingtai, Hebei 054001 China; 8 Department of Infectious Disease, Friendship Hospital, Beijing, 100029 China; 9 Department of Liver Disease, Tianjin Second People’s Hospital, Tianjin, 300192 China; 10 Department of Traditional and Western Medical Hepatology, The Third Hospital of Hebei Medical University, Shijiazhuang, Hebei 050051 China; 11 Department of Liver Disease, Shijiazhuang Fifth Hospital, Shijiazhuang, Hebei 050021 China; 12 Research Center for Technologies in Nucleic Acid-Based Diagnostics, Changsha, Hunan 410205 China; 13 Department of Hepatopancreatobiliary Disease, Beijing Tsinghua Changgung Hospital, School of Clinical Medicine, Tsinghua University, Beijing, 102218 China

**Keywords:** Nucleos(t)ide analog, Cessation, Hepatitis B surface antigen, Hepatitis B core-related antigen, Hepatitis B virus RNA

## Abstract

**Background:**

Since hepatitis B surface antigen (HBsAg) loss is rarely achieved with nucleos(t)ide analog (NA) treatment, most patients require life-long NA treatment. Previous studies have shown that some patients remain virologically responsive even after NA cessation. However, there is still controversy surrounding whether NA discontinuation increases the HBsAg loss rate. Therefore, this study aimed to assess the cumulative rate of HBsAg loss and identify the predictors of HBsAg loss after NA discontinuation.

**Methods:**

This multicenter prospective study included HBV e antigen (HBeAg)-positive patients without cirrhosis from 12 hospitals in China who met the inclusion criteria. The enrolled patients stopped NA and were followed up with clinical and laboratory assessments every 3 months for 24 months after NA cessation or until clinical relapse (CR) occurred.

**Results:**

Overall, 158 patients were classified into two groups. Group A included patients with HBsAg positivity at NA cessation (n = 139), and Group B included patients with HBsAg negativity at NA cessation (n = 19). In Group A, the 12-month and 24-month cumulative rates of HBsAg loss were4.3%and 9.4%, respectively. End of treatment (EOT) HBsAg (hazard ratio (HR) = 0.152, *P* < 0.001) and EOT hepatitis B core-related antigen (HBcrAg) (HR = 0.257, *P* = 0.001) were associated with HBsAg loss. The areas under the receiver operating characteristic curves for EOT HBsAg and HBcrAg levels were 0.952 (*P* < 0.001) and 0.765 (*P* < 0.001), respectively. Patients with EOT HBsAg ≤ 135 IU/mL (59.2% vs. 1.3%, *P* < 0.001) or HBcrAg ≤ 3.6 logU/mL (17% vs. 5.4%, *P =* 0.027) had a higher 24-month cumulative HBsAg loss rate. In Group B, none of the patients experienced virological relapse after NA cessation. Only 1 (5.3%) patient had HBsAg reversion.

**Conclusions:**

EOT HBsAg ≤ 135 IU/mL or HBcrAg ≤ 3.6 logU/mL can be used to identify patients with a higher likelihood of HBsAg loss after NA cessation. Patients with HBsAg negativity after NA cessation have favorable clinical outcomes, and HBsAg loss was durable in most cases.

## Background

Viral suppression using nucleos(t)ide analog (NA) improves the prognosis of patients with chronic hepatitis B (CHB) and reduces the risk of hepatocellular carcinoma (HCC). However, NA treatment cannot eliminate intrahepatic covalently closed circular DNA (cccDNA) or achieve hepatitis B surface antigen (HBsAg) seroclearance [1, 2]. Interestingly, recent studies have shown that a proportion of patients maintained virological response even after discontinuing NA, suggesting that cessation may increase the rate of HBsAg loss [1, 3]. For example, in the DARING-B study following 57 patients who discontinued NA, the cumulative rates of HBsAg loss were 5%, 16%, and 25% at 6, 12, and 18 months, respectively [4]. Another retrospective study from Taiwan, involving 691 hepatitis B e antigen (HBeAg)-negative CHB patients who stopped treatment, revealed a 13% HBsAg loss after 6 years of treatment, which was significantly higher than that during treatment [1].

It is important to identify predictors that can determine which patients are more likely to experience HBsAg loss after NA cessation. Some studies have reported that patients with lower end-of-treatment (EOT) HBsAg levels have a higher chance of achieving HBsAg loss after NA discontinuation [5, 6]. However, other studies have failed to find a significant association [7, 8]. Evaluating the predictive value of other HBV serum markers, such as HBV core-related (HBcrAg) and HBV RNA, may be of interest, although data are scarce [6, 9]. Furthermore, several studies have explored the predictive power of combining these HBV markers.

However, research findings have indicated that NA-induced HBsAg loss shows similar durability to spontaneous HBsAg loss and is associated with favorable clinical outcomes [10, 11]. Several studies have shown that relapses are absent in patients with CHB who discontinue NA after HBsAg loss [12]. However, other studies have reported rates of HBsAg seroreversion ranging from 4.8 to 11.7% at 3 years after NA discontinuation [10, 11]. Whether NA-treated patients with HBsAg loss can safely stop NA treatment has not been well established.

Therefore, we conducted this prospective study to address two key questions: (1) the incidence and predictors of HBsAg loss after NA cessation and (2) whether HBsAg loss is durable in patients with HBsAg negativity upon NA cessation.

## Methods

### Patients

A total of 158 initially HBeAg-positive CHB patients without cirrhosis discontinued NA treatment and were followed up from January 2017 to December 2020 in 12 hospitals in Beijing, Tianjin, and Hebei provinces in China. The patients were classified into two groups according to their HBsAg status after NA cessation. Group A included patients with HBsAg positivity at NA cessation (n = 139), while Group B included patients with HBsAg negativity at NA cessation (n = 19).

The inclusion and exclusion criteria and follow-up schedule were described in our previous study [13]. Briefly, all patients met the NA cessation criteria, which included undetectable serum HBV DNA, normal serum alanine aminotransferase (ALT) levels, and HBeAg seroconversion for at least 3 years. Additionally, the duration of NA treatment was more than 4 years, according to the Chinese guidelines for the prevention and treatment of chronic hepatitis B [14]. All patients discontinued NA upon entering this study and were followed-up every 3 months for 24 months or until clinical relapse (CR). Retreatment was initiated if CR was observed, defined as ALT level > 2 ULN along with an HBV DNA level of > 2000 IU/mL, which was considered virological relapse (VR). Consolidation therapy was defined as the duration of treatment from the first report of HBeAg seroconversion until NA cessation [13].

This study was approved by the Institutional Review Board of Peking University People’s Hospital (2017PHB001-01), and written informed consent was obtained from all the patients.

### Biochemical and virological test

Serum ALT levels were tested at local laboratories with the ULN set at 40 U/L. HBV DNA, HBsAg, and anti-HBs were assessed in a central laboratory located at Peking University People’s Hospital. All methods were performed in accordance with the relevant guidelines and regulations. HBV DNA was quantitatively detected using the Roche COBAS TaqMan HBV Test, with a lower detection limit of 20 IU/mL. Serum HBsAg and anti-HBs levels were quantified using an automated chemiluminescent assay (Architect I2000SR; Abbott). Range of the HBsAg test was 0.05–250 IU/mL. If the HBsAg level was > 250 IU/mL, serial dilutions from 1:100 to 1:1000 were performed. An anti-HBs level ≥ 10 mIU/mL was considered positive.

HBV RNA was isolated using the Diagnostic Kit for Hepatitis B virus pgRNA (PCR-Fluorescence Probing) (Hotgen Biotech, Beijing, China) and detected by quantitative real-time polymerase chain reaction (PCR) using an ABI Prism 7500 Real-time PCR System (ABI, USA). The lower detection limit was 300 copies/mL.

A chemiluminescent enzyme immunoassay (Lumipulse G HBcrAg assay) was employed to measure serum HBcrAg levels using a Lumipulse G1200 analyzer manufactured by (Fujirebio, Japan). The lower limit of quantification was 3 logU/mL. In cases where the sample concentration was > 7 logU/mL, dilution and subsequence retesting were performed.

### Statistical analysis

Categorical variables were expressed as numbers (percentages), while continuous variables were summarized as medians (interquartile ranges). The HBsAg and HBV RNA levels were logarithmically transformed for statistical analysis. To analyze the differences between groups, either the χ2 test or the Student *t* test was deployed. Kaplan-Meier analyses were used to calculate the cumulative rates of HBsAg loss, which were compared using the log-rank test. Cox regression analysis was performed to assess predictors of off-treatment HBsAg loss. The accuracy of the serum markers in predicting HBsAg loss was assessed using the area under the receiver operating characteristic (AUROC) curve. Statistical significance was defined as a two-tailed *P* value < 0.05. Statistical analyses and representations were performed using IBM SPSS software version 26.0, while Graphical analyses and representation of data were conducted using GraphPad Prism 7.0 software.

## Results

### Group A: patients with HBsAg positive when NA cessation

#### Characteristics of patients at the start of treatment and end of treatment

Overall, 139 patients with HBsAg positivity at NA cessation were classified into Group A. All enrolled patients were of Asian descent, with 58.3% being male, and the median age was 36 years. All subjects were treated with NA for an average duration of 6.4 (4.7–8.6) years. The duration of undetectable HBV DNA and HBeAg seroconversion before NA cessation was 5.8 (4.3–7.8) years and 4 (3.5–5.8) years, respectively. The range of HBsAg ranged from 0.05 to18512.6 IU/mL. Among the patients 22 (15.8%) had EOT HBsAg levels < 100 IU/mL, and 39 (28.1%) had levels between 100 and 1,000 IU/mL.The 12-month cumulative rates of VR, CR, HBeAg reversion, and HBsAg loss were 38.8%, 15.1%, 8.6%,and 4.3%, respectively, and the corresponding 24-month cumulative rates were 50.4%, 24.5%, 11.5%,and 9.4%, respectively [13]. Patients with HBsAg loss (n = 13) had a lower percentage of a family history of hepatitis B (23.1% VS 53.2%, *P* = 0.039). The proportion of patients with HBsAg-loss was higher among males (*P* = 0.043), and they were older following NA cessation (*P* < 0.001). Significant differences in EOT HBsAg, HBcrAg, and HBV RNA levels were observed between patients with and without HBsAg loss. Patients with HBsAg loss had lower HBsAg (median 0.7 vs. 3.2 log_10_IU/mL, *P* < 0.001) and HBcrAg (median 3.2 vs. 3.8 logU/mL, *P* = 0.001) levels at the end of treatment. Simultaneously, a higher proportion of them had negative HBV RNA (92.3 vs. 69%, *P* = 0.012) (Table 1).

**Table 1:** Characteristics of Group A patients at the start of treatment and end of treatment

	All(n = 139)	HBsAg loss(n = 13)	No HBsAg-loss(n = 126)	*P* Value
Start of treatment				
HBV DNA,log_10_IU/mL	5.9(5.4–6.8)	5.7(5.4–6.9)	5.9(5.2–6.7)	0.498
HBsAg,log_10_IU/mL	3.5(3.1–3.8)	3.8(3.3-4.0)	3.5(3.1–3.8)	0.747
Family history of hepatitis B	70(50.4%)	3(23.1%)	67(53.2%)	0.039
Family history of HCC	13(9.4%)	1(7.7%)	12(9.5%)	0.350
**End of treatment**				
Age,y	36(31–45)	55(47.5–61.5)	35(31-40.3)	< 0.001
Male gender	81(58.3%)	11(84.6%)	70(55.6%)	0.043
Body mass index,Kg/m^2^	23(21.1–24.8)	23.4(22–27)	23(21-24.8)	0.067
Current antiviral treatment regimen				0.315
Entecavir	99(71.2%)	7(53.8%)	92(73%)	
Tenofovir	16(11.5%)	2(15.4%)	14(11.1%)	
others	24(17.3%)	4(30.8%)	20(15.9%)	
Treatment duration,y	6.4(4.7–8.6)	7.8(6.8–9.4)	6.1(4.5–8.3)	0.089
Duration of undetectable HBV DNA,y	5.8(4.3–7.8)	7.2(6-8.5)	5.2(3.8–7.8)	0.091
Duration of HBeAg seroconversion,y	4(3.5–5.8)	4.7(3.8–6.5)	4(3.5–5.7)	0.616
ALT, U/L	21(13–32)	21(12–31)	21(15–33)	0.686
Liver stiffness,kPa	4.8(4.1–5.8)	4.4(4.1–5.5)	4.8(4.1–5.8)	0.494
CAP,dB/m	218(191–258)	219(181–316)	217(190–254)	0.521
HBsAg,log_10_IU/mL	3.2(2.6–3.6)	0.7(-1.0-2.0)	3.2(2.8–3.7)	< 0.001
HBV RNA,log_10_copies/mL	0(0–2)	0	0(0-2.2)	0.012
Negative HBV RNA	99(71.2%)	12(92.3%)	87(69%)	
HBcrAg,logU/mL	3.8(3.3–4.2)	3.2(2.4–3.7)	3.8(3.4–4.2)	0.001

Data are expressed as median values (interquartile ranges) or no.(%) of individuals

HCC,hepatocellular carcinoma; CAP,the controlled attenuation parameter; HBV,hepatitis B virus; HBsAg,hepatitis B surface antigen; HBcrAg,hepatitis B core-related antigen.

### Kinetics of HBsAg, HBcrAg, and HBV RNA after NA cessation

**Fig. 1:**
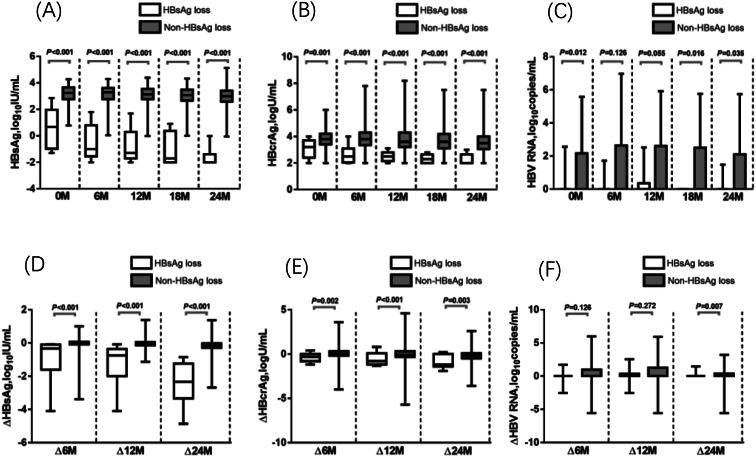
(**A**)-(**C**), Serum hepatitis B surface antigen (HBsAg), hepatitis B core-related antigen (HBcrAg) and hepatitis B virus (HBV) RNA at end of treatment, 6 months, 12 months, 18 months and 24 months after treatment cessation in Group A patients with HBsAg loss or without HBsAg loss. (**D**)-(**F**), Comparison of HBsAg, HBcrAg and HBV RNA change from end of treatment to 6 months, 12 months and 24 months after treatment cessation in Group A patients with HBsAg loss or without HBsAg loss. The box plots showed median, interquartile range and absolute range

As shown in Fig. 1 (A)–(C), significant differences were observed in the levels of HBsAg and HBcrAg between patients with and without HBsAg loss. Patients with HBsAg loss had lower HBsAg (*P* < 0.001) and HBcrAg (*P* < 0.001 or *P* = 0.001) levels at the end of treatment and at 6, 12, 18, and 24 months after NA cessation, respectively. Notably, significant differences in HBV RNA levels were found at the end of treatment and at 18 and 24 months after NA cessation but not at 6 and 12 months after NA cessation.

As shown in Fig. 1 (D)–(F), rapid decreases in HBsAg (*P* < 0.001) and HBcrAg (*P* < 0.001 or *P* = 0.001) levels were observed from baseline to 6, 12, and 24months after NA discontinuation in patients with HBsAg loss. However, no significant differences were found inΔHBV RNA from baseline to 6, 12, and 24months after NA cessation (*P* > 0.05).

### Predictors forHBsAg loss after NA cessation

Changes in HBsAg levels after NA cessation were reported in our previous study [13]. Cox regression analysis was conducted to identify predictors of HBsAg loss after NA cessation. Neither the treatment duration nor the consolidation treatment duration was associated with HBsAg loss. Age (hazard ratio [HR], 1.138; 95% confidence interval [CI], 1.073–1.207; *P* < 0.001), EOT HBsAg level (HR, 0.152; 95% CI, 0.065–0.355; *P* < 0.001), and EOT HBcrAg level (HR, 0.257; 95% CI, 0.113–0.586; *P* = 0.001) were independent predictors of HBsAg loss. Rapid decreases in HBsAg and HBcrAg levels from baseline to 6-month (Δ6m), 12-month (Δ12m), and 24-month (Δ24m) after NA discontinuation were also associated with HBsAg loss (Table 2).

**Table 2:** Predictors for HBsAg loss in Group A patients by Cox regression

Variable	Hazard ratio(95% CI)	*P* Value
Age	1.138(1.073–1.207)	< 0.001
Gender(male vs. female)	0.227(0.048–1.068)	0.061
Treatment duration	1.010(0.998–1.022)	0.097
Treatment duration(< 6 vs. ≥ 6 y)	0.14(0.018–1.112)	0.063
Consolidation treatment duration	1.005(0.985–1.026)	0.614
Consolidation treatment duration(< 5 vs. ≥ 5 y)	0.717(0.221–2.333)	0.581
HBV DNA at start of treatment,log_10_IU/mL	1.193(0.718–1.983)	0.496
EOT HBsAg(log_10_IU/mL)	0.152(0.065–0.355)	< 0.001
EOT HBV RNA	0.531(0.235–1.199)	0.127
EOT HBcrAg	0.257(0.113–0.586)	0.001
Δ6mHBsAg	0.208(0.076–0.57)	0.002
Δ6mHBcrAg	0.432(0.195–0.956)	0.038
Δ6mHBV RNA	0.745(0.474–1.173)	0.203
Δ12mHBsAg	0.018(0.003–0.127)	< 0.001
Δ12mHBcrAg	0.628(0.407–0.971)	0.036
Δ12mHBV RNA	1(0.999–1.001)	0.720
Δ24mHBsAg	0.043(0.007–0.252)	0.001
Δ24mHBcrAg	0.331(0.147–0.744)	0.007
Δ24mHBV RNA	0.997(0.989–1.005)	0.467

Note. Consolidation treatment duration was defined as the treatment duration after achieving hepatitis B e antigen seroconversion. EOT, end of treatment; HBV,hepatitis B virus; HBsAg,hepatitis B surface antigen; HBcrAg,hepatitis B core-related antigen.

**Fig. 2:**
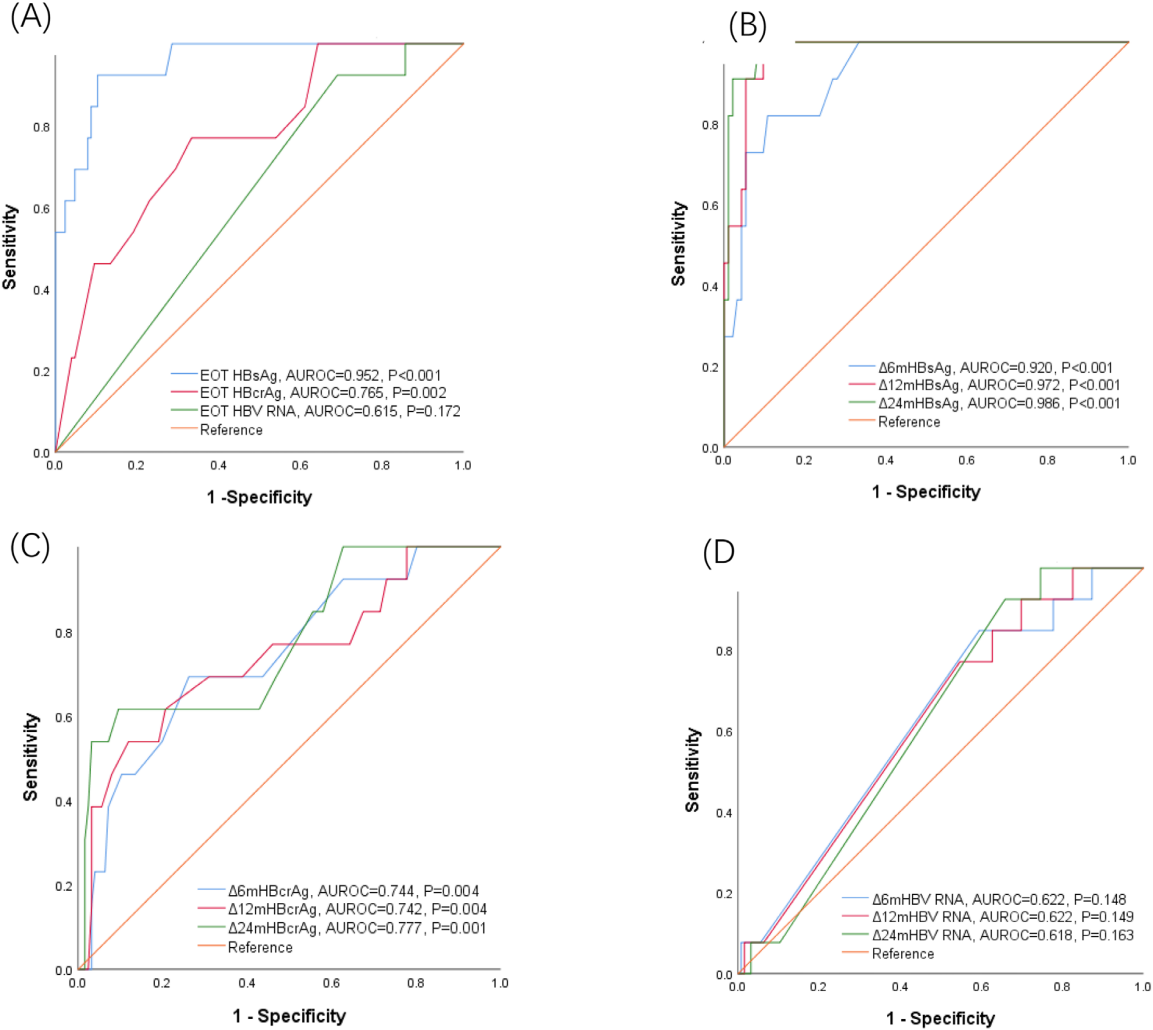
(**A**) Area under the receiver-operating characteristic curves (AUROC) of end of treatment (EOT) hepatitis B surface antigen (HBsAg), hepatitis B core-related antigen (HBcrAg) and hepatitis B virus (HBV) RNA for predicting HBsAg loss in Group A patients. (**B**) ΔHBsAg as a predictor of HBsAg loss at 6 months, 12 months and 24 months after treatment cessation. (**C**) ΔHBcrAg as a predictor of HBsAg loss at 6 months, 12 months and 24 months after treatment cessation. (**D**) ΔHBV RNA as a predictor of HBsAg loss at 6 months, 12 months and 24 months after treatment cessation

To further assess the ability of EOT HBsAg, EOT HBcrAg, EOT HBV RNA, ΔHBsAg, ΔHBcrAg, and ΔHBV RNA levels to predict HBsAg loss, AUROC values for each parameter were calculated (Fig. 2). An EOT HBsAg level of 135 IU/mL had the highest Youden’s index with an AUROC value of 0.952 (95% CI 0.906–0.998, *P* < 0.001), sensitivity of 0.923, and specificity of 0.897. The AUROC values of ΔHBsAg at 6 and 12 months were 0.920 (95% CI 0.852–0.988, *P* < 0.001) and 0.972 (95% CI 0.942-1, *P* < 0.001), respectively. An EOT HBcrAg of 3.6 IU/mL had the maximum Youden’s index with an AUROC value of 0.765 (95% CI 0.635–0.895, *P =* 0.002), sensitivity of 0.769, and specificity of 0.667. The AUROC value of ΔHBcrAg at 6 months and 12 months were 0.744 (95% CI 0.603–0.884, *P =* 0.004), 0.742 (95% CI 0.586–0.898, *P =* 0.004), respectively. EOT HBV RNA and ΔHBV RNA levels failed to predict HBsAg loss after NA cessation at 6, 12, and 24 months (*P* > 0.05).

### Stratified analysis of cumulative HBsAg loss rate

**Fig. 3:**
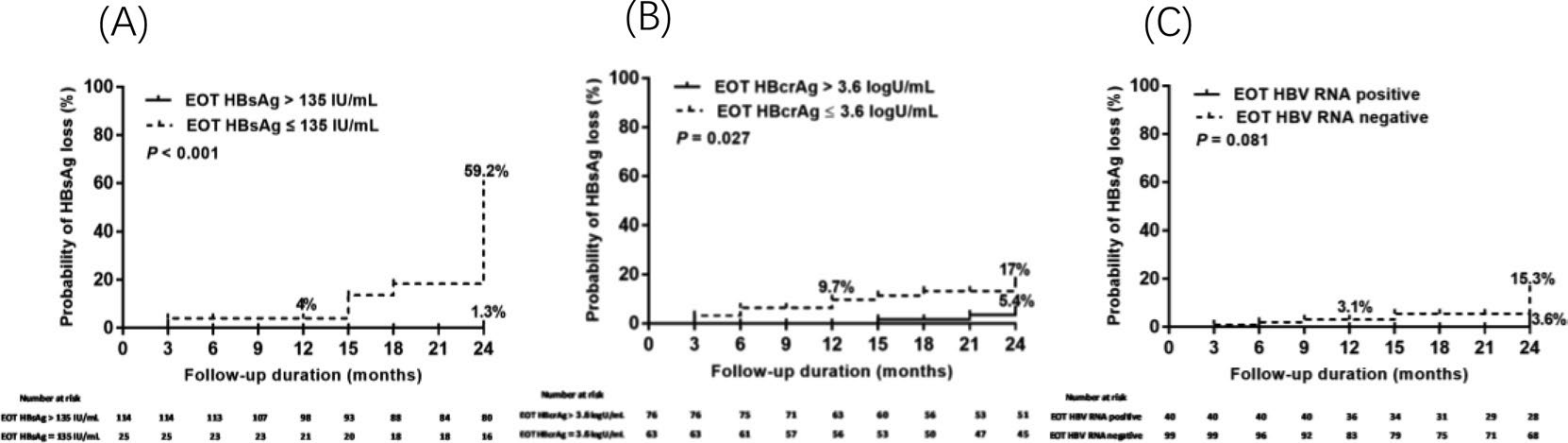
Cumulative incidences of hepatitis B surface antigen (HBsAg) loss stratified by end of treatment (EOT) HBsAg, hepatitis B core-related antigen (HBcrAg) and hepatitis B virus (HBV) RNA in Group A patients

As shown in Fig. 3, the cumulative probabilities of HBsAg loss were stratified based on EOT HBsAg, HBcrAg, or HBV RNA levels. Over a 24 months period after NA cessation, cumulative rates of HBsAg loss in patients with EOT HBsAg ≤ 135 IU/mL and > 135 IU/mL were 59.2% and 1.3%, respectively (*P* < 0.001, Fig. 3A). Stratifying the cumulative incidences of HBsAg loss by EOT HBcrAg ≤ 3.6 logU/mL and > 3.6 logU/mL revealed rates of 17% and 5.4%, respectively (*P* = 0.021, Fig. 3B). No significant difference was observed between patients with EOT HBV RNA-negative and EOT HBV RNA-positive patients (15.3% vs.3.6%, *P* = 0.081; Fig. 3C).

### Group B: patients with HBsAg negative when NA cessation

#### Characteristics of patients at the start of treatment and end of treatment

A total of 19 patients (68.4% male; median age, 60 years) with HBsAg negative when NA cessation were classified into Group B. All patients were treated for a duration of 6.8 (5.8–12.8) years with NA. Among them, Nine (47.4%) were treated with entecavir, and 36.8% were treated with non-first-line NA therapy. The duration of undetectable HBV DNA and HBeAg seroconversion before the end of treatment was 6 (4.8–12) years and 4 (3.8–6.1) years, respectively. Following HBsAg loss, NA treatment was continued for 1.8 (1–3) years in all the patients (Table 3).

**Table 3:** Characteristics of Group B patients at the start of treatment and end of treatment

Parameter	Value
Start of treatment	
HBV DNA,log_10_IU/mL	6.1(5.2–6.8)
HBsAg,log10IU/mL	3.3(2.6-4)
Family history of hepatits B	4(21.1%)
Family history of HCC	3(15.8%)
**End of treatment**	
Age,y	60(38–65)
Male gender	13(68.4%)
Body mass index,Kg/m^2^	23.4(22-26.2)
Current antiviral treatment regimen	
Entecavir	9(47.4%)
Tenofovir	3(15.8%)
others	7(36.8%)
Treatment duration,y	6.8(5.8–12.8)
Duration of undetectable HBV DNA,y	6(4.8–12)
Duration of HBeAg seroconversion,y	4(3.8–6.1)
Duration of HBsAg loss,y	1.8(1–3)
Liver stiffness,kPa	4.7(3.8–6.6)
CAP,dB/m	234(197–248)
Anti-HBs,mIU/mL	10.2(2.6–33.5)
HBV RNA,log_10_copies/mL	0(0–0)
Negative HBV RNA	17(89.5%)
HBcrAg,logU/mL	3.8(3.2-4)

Data are expressed as median values (interquartile ranges) or no.(%) of individuals

HCC, hepatocellular carcinoma; CAP,the controlled attenuation parameter; HBV,hepatitis B virus; HBsAg,hepatitis B surface antigen; HBcrAg,hepatitis B core-related antigen.

### Virological relapse, clinical relapse, and HBsAg reversion after NA cessation

The median follow-up duration was 21 (12–24) months. None of the patients achieved VR or CR after NA cessation. Only one patient (5.3%) experienced HBsAg reverse-positive at 6 months. HBV DNA was detectable at 21 months but remained below 2000 IU/mL after NA cessation. Throughout the 24 months follow-up period after NA cessation, this patient exhibited detectable HBV DNA (< 2000 IU/mL) without any elevation in ALT levels.

### Changes in serum anti-HBs, HBcrAg, and HBV RNA levels after NA cessation

**Fig. 4:**
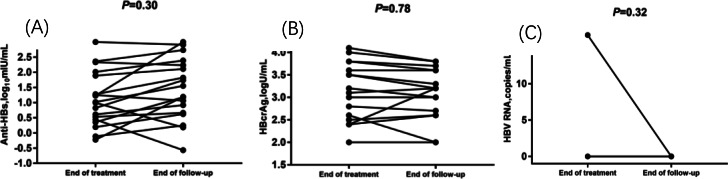
Dynamic changes of anti-HBs, hepatitis B core-related antigen (HBcrAg) and hepatitis B virus (HBV) RNA levels at end of treatment and end of follow-up in Group B patients (n = 18, excluding one patient with HBsAg reversion)

The patient who experienced HBsAg reversion at 6 months after NA cessation exhibited anti-HBs positivity at the end of treatment (10.83 mIU/mL) and at 3 months after NA cessation (12.88 mIU/mL). Among the remaining 18 patients in Group B, 9 patients had positive anti-HBs levels at the end of treatment, which remained positive during follow-up, with a majority showing an increasing trend. Three patients initially tested negative for anti-HBs at the end of treatment but later tested positive after NA cessation. The other six patients consistently tested negative for ani-HBs both at the end of treatment and during follow-up. After the 24-month follow-up, no significant difference in anti-HB levels was found between EOT and the end of the follow-up (Fig. 4A).

Serum HBV RNA was positive in 2 patients after NA cessation, including the patient with HBsAg reversion. Serum HBV RNA levels fluctuated positively in 5 other patients after withdrawal, accompanied by an increasing trend in anti-HBs. In contrast, seven patients with positive serum HBcrAg levels at EOT remained positive after NA discontinuation. Compared to serum HBV RNA, serum HBcrAg levels demonstrated relatively stable fluctuations with minimal changes (Fig. 4BC).

## Discussion

In this prospective multicenter study, we evaluated the HBsAg loss and changes in HBV serological markers after NA cessation. Our results showed that a subset of patients (9.4%) achieved HBsAg loss, and both EOT HBsAg and HBcrAg levels were useful in identifying patients with a higher likelihood of HBsAg loss after NA cessation. Specifically, EOT HBsAg level was the strongest predictor of HBsAg loss, aligning with previous studies [1, 5, 6]. Notably, EOT HBsAg ≤ 135 IU/mL had good predictability for HBsAg loss, offering high sensitivity and specificity. Furthermore, our data also showed that EOT HbcrAg ≤ 3.6logU/mL could effectively predict HBsAg loss after NA discontinuation. This study focused on the outcomes of HBsAg-negative patients following NA cessation, demonstrating that these patients maintained a functional cure and had good long-term prognosis during the 24 months follow-up.

Although the discontinuation of NA remains a matter of debate in clinical practice, NA cessation is recommended in all current treatment guidelines. In this study, we used the Chinese NA cessation criteria, which limit longer treatment and consolidation therapy, to provide more research data for discussing different NA withdrawal criteria. Growing evidence has shown that a significant, albeit small, proportion of patients with CHB achieve HBsAg loss after NA discontinuation. HBsAg loss rates after NA discontinuation vary widely among studies. Our patients demonstrated a relatively low rate of HBsAg loss after cessation of NA treatment. Similar results were reported in a study from Germany [15] and several studies from Asia [1, 16], whereas higher rates of HBsAg loss were found in other studies [5, 17, 18]. It is unclear whether these differences stem from ethnicity or other potential confounding factors. Our current study, comprising exclusively of Asians, suggests that Asian ethnicities may have lower rates of HBsAg loss than non-Asian ethnicities, consistent with Sonneveld’s study [19]. It is difficult to compare the rates of HBsAg loss among studies because of different patients and viral characteristics, and most importantly, different baseline and EOT HBsAg levels. These are remarkable findings compared with the very low HBsAg loss rate during NA treatment. The mechanism underlying HBsAg loss after the cessation of NA treatment remains unclear. Long-term NA treatment has been hypothesized to modulate and restore T-cell responses [20]. The reappearance of HBV replication after NA cessation is considered an essential trigger that may ultimately lead to complete immune control of HBV infection [21].

Berg et al. [22] proposed that NA discontinuation-associated relapse is an integral part of the stop-to-cure approach and ultimately triggers HBsAg loss. After NA cessation, HBV DNA and ALT flares are often transient in patients who relapse. Whether there are, favorable and unfavorable flares in terms of the induction of long-term remission and HBsAg loss and how to distinguish between them is controversial, as is the question of why only a minority of patients achieve a functional cure. However, our data did not support the concept that HBsAg loss occurs more frequently in patients with relapse. In our study, only one patient who lost HBsAg had clinical relapse, and the remaining 12 patients did not have VR or CR. Notably, the occurrence of HBsAg loss was significantly or numerically less frequent in patients with HBV DNA or ALT flares, which is consistent with the findings of other studies [1, 23]. Given the conflicting results in this area, it is important to identify additional markers related to HBsAg loss after the discontinuation of NAs.

HBsAg as a serological indicator of transcriptionally active cccDNA, and has been identified as a reliable predictor of both relapse and HBsAg loss after NA cessation. In most studies, a greater HBsAg decline during treatment and lower EOT HBsAg levels have been associated with a higher likelihood of HBsAg loss after NA discontinuation [1, 16, 19, 24]. However, the use of EOT HBsAg cutoff level as a predictor of HBsAg loss remains controversial. In the DARING-B study, patients with EOT HBsAg levels < 100 IU/mL exhibited a remarkable f HBsAg loss rate of up to 77%, at 18 months after stopping NA treatment [6]. Similarly, in another study involving 411 patients, the 8-year HBsAg loss rate was 69.3% in HBeAg-negative patients with EOT HBsAg < 200 IU/mL, while the 5-year HBsAg loss rate was 47.3% in HBeAg-positive patients with EOT HBsAg < 300 IU/mL [25]. Our results showed that patients with EOT HBsAg ≤ 135 IU/mL had a high rate (59.2%) of HBsAg loss 24 months after NA cessation, whereas patients with EOT HBsAg > 135 IU/mL rarely achieved HBsAg loss (1.3%) (Fig. 3). However, It should be noted that EOT HBsAg levels ≤ 135 IU/mL were only observed in a very small proportion of patients (18%) undergoing long-term NA treatment. Therefore, this criterion alone cannot be relied upon to select patients who are likely to achieve HBsAg loss after stopping NA treatment. Emerging data suggest that lower EOT HBsAg, HBcrAg, and HBV RNA levels are associated with a functional cure. In addition, the combination of two or more viral markers, including EOT HBsAg, HBcrAg, and HBV RNA, have been shown to be more predictive for HBsAg loss after NA treatment cessation [26, 27].

Serum HBcrAg is another novel marker that strongly correlates with intrahepatic cccDNA. Even when serum HBV DNA is undetectable or HBsAg loss is achieved, HBcrAg may be detectable for a long time, enabling it to reflect the HBV status after NA cessation [28–30]. Emerging data explore whether EOT HBcrAg levels are associated with different outcomes. A study by Hsu et al. [31] in Taiwan showed that higher HBcrAg levels were associated with an increased risk of clinical relapse. Another study by Papatheodoridis et al. [4] did not support an association between HBcrAg levels and clinical relapse. It remains unclear whether HBcrAg is a good marker for identifying who can stop NA. However, few studies have investigated the predictive ability of EOT HBcrAg levels for HBsAg loss after NA cessation. Sonneveld’s study showed that undetectable HBcrAg levels at the end of treatment were associated with higher rates of HBsAg loss [21]. In our study, patients with HBsAg loss had lower HBcrAg (*P =* 0.001) compared to those without HBsAg loss. The results of the Cox regression analysis showed that EOT HBcrAg (HR, 0.257; 95% CI, 0.113–0.586; *P* = 0.001) was an independent predictor of HBsAg loss (Table 2). Among our patients, those with EOT HBcrAg ≤ 3.6 logU/mL had a high rate (17%) of HBsAg loss at 24 months after NA cessation (Fig. 3). Overall, it remains unclear whether EOT HBcrAg level could serve as a reliable marker for selecting patients who can achieve HBsAg loss after NA cessation. Therefore, further studies are needed to address this issue. As the lower limit of quantification was 3 logU/mL, the limitation of detection may explain why the influence of EOT HBcrAg on HBsAg loss was not obvious in all patients. In the future, EOT HBcrAg might be a useful marker to predict HBsAg loss after NA deletion if the HBcrAg assay can detect lower values. However, it should be noted that serum HBcrAg may become undetectable in HBeAg-negative patients undergoing long-term NA therapy, thus limiting its predictive power [6, 9].

Recently, serum HBV RNA has emerged as a potential biomarker for HBV replication, capable of detection even after NA discontinuation has been studied as an emerging biomarker HBV replication was researched as an emerging biomarker for HBV replication, which could be detectable even after NA discontinuation. In most studies, low or undetectable serum HBV RNA levels have been associated with a reduction in relapse [9, 32, 33]. In our previous study, patients with negative EOT HBV RNA had a significantly lower rate of virological relapse 24 months after NA cessation (39.4% vs. 77.5%, *P* < 0.001) [13]. In this study including the same populations, EOT HBV RNA (*P* = 0.012) was observed in patients with and without HBsAg loss (Table 1). However, EOT HBV RNA level (HR, 0.531; 95% CI, 0.235–1.199; *P* = 0.127) was not an independent predictor of HBsAg loss (Table 2). The detection of HBV RNA provides ancillary information and highlights the limitations of HBsAg and HBV DNA detection in predicting relapse after NA cessation. Until now, studies on the correlation between HBV RNA and HBsAg loss have been scarce and urgently needed.

In this study, decreased levels of HBsAg, HBcrAg, and HBV RNA from the EOT to 6, 12, 18, and 24 months after NA cessation were defined as ΔHBsAg, ΔHBcrAg, and ΔHBV RNA, respectively, to predict HBsAg loss in HBeAg-positive patients. The results showed that rapid decrease of HBsAg and HBcrAg from baseline to 6-month (Δ6m), 12-month (Δ12m), and 24-month (Δ24m) after NA cessation were associated with HBsAg loss, but ΔHBV RNA failed to predict the HBsAg loss after NA cessation. We explored operable predictive factors that could guide NA discontinuation, therefore, changes in these HBV markers after NA cessation cannot be used to select patients who can benefit from discontinuing NA treatment. However, monitoring the decline in HBsAg at 6 and 12 months would be an effective method for selecting patients who can achieve HBsAg loss by adhering to NA cessation.

Our study included 19 patients who achieved HBsAg loss when NA treatment was discontinued. It remians unclear whether consolidation treatment after HBsAg loss is required before NA discontinuation. In our study, NA treatment was continued for 1.8 (1–3) years after HBsAg loss in all patients (Table 3). Other studies have reported median consolidation duration ranging from 7 to 32.5 months [26, 34]. A study from Spain showed that consolidation duration did not affect the persistence of HBsAg loss or the development of anti-HBs after NA cessation [34]. Because of the difficulty associated with conducting a prospective randomized study, a consolidation duration of up to 12 months has been proposed. The development of anti-HBs after HBsAg loss may reflect more profound host immune pressure, leading to more effective control of HBV replication [35]. In this study, anti-HBs were detected at the time of treatment discontinuation in 52.6% of the patients, and no significant difference in anti-HB levels was observed between EOT and 24-month after NA cessation (Fig. 4A). Only one patient with HBsAg reversion 6 months after NA cessation was anti-HBs-positive at the end of treatment. The impact of anti-HBs development is uncertain, however, it appears that the absence of anti-HBs does not confer a poorer prognosis [26, 36]. Only two studies included patients with HBsAg loss at the time of NA cessation, and the number of patients included was relatively small [9, 37]. Therefore, although the number of patients with HBsAg loss in this study was too small to draw conclusions, the results are representative and valuable for future studies. This study showed that patients with HBsAg loss during NA treatment had a good long-term prognosis after NA cessation during a follow-up period of 24 months.

Our study has some limitations. First, only initially HBeAg-positive patients were included in this study. Therefore, further investigation is needed to determine whether our findings can be applied to initially HBeAg-negative patients. Second, to avoid significant liver injury, patients were retreated once a clinical relapse occurred. Therefore, comparing the rates of off-treatment HBsAg loss between patients with and without retreatment was not possible. Third, only Asian patients were included in this study, and their application to Western patients requires further investigation.

## Conclusion

In conclusion, decisions regarding NA cessation should be made with a thorough understanding of both medical and patient-related factors. Close follow-ups and strict patient adherence are required to reduce the risk of significant disease relapse and liver failure after NA discontinuation. The wider use of biomarkers that reflect the transcriptional activity of cccDNA in HBV DNA-undetectable settings, such as HBsAg, HBcrAg, and HBV RNA, would help physicians in identifying patients with a lower risk of relapse and a higher likelihood of HBsAg loss, thus enabling them to discontinue NA treatment. According to our findings, patients with HBsAg ≤ 135 IU/mL or HBcrAg ≤ 3.6 logU/mL who discontinue NA treatment may have a higher chance of achieving off-treatment HBsAg loss. Furthermore, patients with HBsAg negativity at NA cessation exhibited favorable clinical outcomes, and HBsAg loss was durable in most cases during 24 months follow-up.

## Data Availability

The datasets used and/or analyzed during the current study are available from the corresponding author on reasonable request.

## Abbreviations

ALT: Alanine aminotransferase
AUROC: Area under thereceiver operating characteristic
cccDNA: Covalently closed circular DNA
CHB: Chronic hepatitis B
CI: Confidence interval
CR: Clinical relapse
EOT: End of treatment
HBcrAg: Hepatitis B core-related antigen
HBeAg: Hepatitis B e antigen
HBV: Hepatitis B virus
HBsAg: Hepatitis B surface antigen
HCC: Hepatocellular carcinoma
HR: Hazard ratio
NA: Nucleos(t)ide analogue
VR: Virological relapse
